# Acupuncture for pediatric chronic pain: a systematic review

**DOI:** 10.1016/j.jped.2024.03.013

**Published:** 2024-04-30

**Authors:** João Roberto Bissoto, José Udevanier Rebouças da Silva Júnior, Gabrielle Pignoli Alvares, Flávia H. Santos, Claudio Arnaldo Len

**Affiliations:** a Departamento de Pediatria, Grupo de Reumatologia Pediátrica, Universidade Federal de São Paulo, São Paulo, SP, Brazil; b Departamento de Ortopedia e Traumatologia, Grupo de Acupuntura, Universidade Federal de São Paulo, São Paulo, SP, Brazil; c Departamento de Psicologia, Campus Bauru, Universidade Estadual Paulista Julio de Mesquita Filho, Bauru, SP, Brazil; d School of Psychology, University College Dublin, Dublin, Ireland

**Keywords:** Acupuncture, Chronic pain, Child, Adolescent, Pain management

## Abstract

**Objectives:**

To survey, analyze and discuss the scientific evidence supporting the use of acupuncture and related techniques in the management of chronic pain in the pediatric population.

**Sources:**

A survey of databases (MEDLINE, Scopus and Scielo) was carried out with search strategies, following the PRISMA statement, without limits on publication dates and languages. Clinical studies (clinical trials, single-arm, and case series) were accepted for review if they included participants aged up to 22 years. Study quality was assessed by MMAT, and the randomized clinical trial was analyzed under the STRICTA criteria.

**Summary of the findings:**

2369 articles were retrieved. After excluding repetitions, 1335 underwent the initial selection. Only 16 articles were selected for full reading, of which 5 were included in the review, being two case series, two single-arm studies, and one randomized clinical trial. The articles were considered of good quality by the adopted criteria.

**Conclusion:**

The analyzed studies showed important clinical results such as the reduction of pain intensity, and improvement in school attendance and social life. However, there are many limitations in study design and sample size. Therefore, there is weak evidence to support the use of acupuncture in the context of pediatric chronic pain, but the positive results reinforce the need for further investigation of the topic with the conduct of larger and well-designed studies, to obtain more data and greater scientific conviction of the findings.

## Introduction

Chronic pain, characterized as pain stemming from various causes that lasts or recurs for more than three months,^1^ is a health condition that significantly diminishes quality of life. Its classifications include primary chronic pain, pain related to cancer, neuropathic pain, trauma-related pain (including postoperative pain), orofacial pain, secondary abdominal pain, and headaches.^1^ Children and teenagers often experience specific challenges such as hindrances in academic progress and interpersonal relationships. Furthermore, there are notable economic repercussions and impacts on family dynamics.^2^^,^^3^

Despite the high prevalence of chronic pain, more than 30 % of pediatric cases,^3^ there is a lack of conclusive studies on the proper management of this condition. The existing ones point to the difficulty of clinical management, due to the multiple functioning areas affected by the desease.^4^, ^5^, ^6^ Current evidence suggests that the best approach is an interdisciplinary one, led by specialized teams, composed of physicians, physiotherapists, psychologists, and other professionals. This integrated work promotes changes in different aspects of the patients' lives. Therefore, it modulates the mechanisms of perpetuation and amplification of pain, not just its causal factors.^4^

These physiological mechanisms involved in the modulation of chronic pain are not yet well established, however, studies point out that the maintenance of symptoms beyond their initial stimulus is related to the reduction of the hypothalamic-pituitary-adrenal axis or to the increase in the sympathetic response to stress.^7^ These changes result in sensitization of the pain transmission and interpretation pathways, allowing for the chronicization of the pain. In addition, many chronic pain conditions do not present measurable tissue damage, which strengthens the hypothesis that there are mechanisms of interaction between neurotransmitters and mediators that are not yet understood.^7^

When addressing pain in pediatric patients, it's essential to tailor assessment and management strategies based on age and cognitive development to ensure optimal care^8^. Utilizing appropriate tools for gauging pain intensity is crucial, as it informs treatment decisions. Additionally, pediatric pain often coincides with anxiety and distress, underscoring the importance of a comprehensive approach that incorporates both pharmacological and non-pharmacological therapies.^8^

The management of chronically ill children is also made difficult by their frequent use of complementary health practices, reaching up to 50 % of this population,^9^ even though the level of scientific evidence supporting this use is incipient and controversial. In this sense, Integrative Medicine addresses this problem and seeks to offer an evidence-based practice that meets the patients' demand for more dynamic and comprehensive care.^9^

Acupuncture is a major exponent of this modality and is being studied for the treatment of various health conditions,^10^ in addition to the fact that many physiological mechanisms involved in its therapeutic action are already known.^11^^,^^12^ This allows professionals to practice it based on scientific evidence, acting safely and effectively in the clinical management of their patients.

Pain is the largest area of research of acupuncture, with well-designed studies on the neural mechanisms involved and its clinical efficacy, the most relevant being the release of endogenous opioids at the spinal cord.^11^ In the adult population, acupuncture is already well established as a technique for controlling chronic pain,^13^ for example, headache, pelvic pain, fibromyalgia, and postoperative pain.^10^ However, in the field of pediatrics, the number of works is still reduced, and the scientific evidence is not well established,^9^ which may justify the scarce inclusion of this technique in pediatric pain centers.

One of the difficulties in building scientific evidence on acupuncture has its roots in its history, as there are many techniques and schools of thought, and this lack of standardization becomes a barrier to the consolidation of systematic studies. For example, there are studies that use Chinese systemic acupuncture, Chinese and Japanese scalp acupuncture, and Japanese systemic acupuncture, among other variations.

Moreover, within Chinese Medicine there are other techniques that are commonly used as complements of acupuncture treatments, such as 1. acupressure and moxibustion (other forms of stimulating the acupoints, the first being manual pressure, and the latter being heat stimuli); 2. cupping, which consists in producing a vacuum that stretches the skin and fascia; 3. auricular therapy, a form of stimulating specific points in the ear that produces systemic effects.

Therefore, it is essential to explain the technique and the rationality used when conducting scientific work on acupuncture^14^. Moxibustion, acupressure, and auricular therapy are of special interest in the field of pediatrics, as they are non-invasive techniques and therefore better accepted by families.

### Acupuncture for pain

The analgesic effects of acupuncture can be divided into three categories: local, segmental, and central or systemic.^15^ The local mechanism is based on the axonal reflex triggered by the pseudounipolar neuron, which activates a response mediated by neuropeptides, such as Calcitonin Gene-Related Peptide, by modulating the release of nitric oxide, adenosine, Tumor Necrosis Factor, as well as activating mast cells and fibroblasts, resulting in tissue regeneration and control of the damaging inflammatory response.^16^

There is compelling evidence indicating that acupuncture's primary mechanism of action in pain management operates segmentally within the spinal cord.^11^ This is achieved through interconnections between dermatomes, sclerotomes, myotomes, and viscerotomes, whereby acupuncture points commonly used for pain treatment are associated with the same level of innervation as the affected site. Numerous studies have demonstrated that this effect is a result of hyperpolarization of the posterior horn of the spinal cord, which inhibits the release of pain-propagating mediators.^15^ This mechanism primarily involves the stimulation of inhibitory cells that release enkephalins in the gelatinous substance, thereby impeding the spread of painful stimuli. Additionally, it has been observed that varying frequencies of electrical stimulation, as seen in electroacupuncture, induce the release of specific endogenous opioids in the central nervous system.^17^

Other studies have shown that the acupuncture stimulation activates areas of the brain responsible for the descending inhibitory pain pathways, as well as areas of interpretation and modulation of the painful stimulus,^15^^,^^18^ which demonstrates the analgesic effects of this technique even when applied outside the spinal segment corresponding to the pain site. There is also evidence that the neural stimulation promoted by acupuncture activates immunomodulation pathways mediated by the Vagus nerve and the dorsal sympathetic ganglia.^16^

In clinical studies, acupuncture has demonstrated its efficacy in chronic pain management in the adult population. An evidence map constructed by Allen et al.^10^ gathered data from systematic reviews and showed that there is good quality evidence for the use of acupuncture in cases of chronic pain due to fibromyalgia, migraine and other headaches, postoperative pain, chronic pelvic pain, and chronic musculoskeletal pain. The authors analyzed the interventions and control groups but didn't discuss different acupuncture techniques or styles.

The increasing acceptance of acupuncture for managing chronic pain in adults is evident through its inclusion in the National Institute for Health and Care Excellence guideline for chronic pain.^13^ Furthermore, recent research is shedding light on acupuncture's wide-ranging effects on pain propagation mechanisms and inflammatory response modulation.^15^^,^^17^^,^^19^

As acupuncture's clinical relevance grows, so does concern about safety and adverse effects. When administered by a trained professional, acupuncture appears to be generally safe, with mild complications such as superficial bleeding, pain at the needled site, and temporary irritability during sessions being reported.^20^

Given the imperative for improved chronic pain management in children and teenagers due to its multifaceted impact on their lives,^2^^,^^4^^,^^5^ and the lack of evidence supporting acupuncture's use in this context, the authors conducted a systematic review to evaluate its efficacy in pediatric chronic pain management.

The authors aimed to gather clinical data on the usage of acupuncture techniques in chronic pediatric pain management (by collecting pain intensity and frequency scores). Besides, knowing the multiple styles of acupuncture, and the variable pattern in the number and frequency of the sessions, the authors collected and discussed this information. In addition, the impact of this treatment on the quality of life of the subjects, and its safety (side effects and complications of the procedure).

## Methods

The protocol for this study was registered with the PROSPERO (International Prospective Register of Systematic Reviews) network of systematic reviews, under the number CRD42022303021 and followed the PRISMA statement (Preferred Reporting Items for Systematic Reviews and Meta-Analyses).^21^

The search was conducted in the MEDLINE, Scopus and Scielo databases. The first survey in these databases took place in September 2022 and was updated in April 2023. The search logic procedure (Table 1) was carried out in stages so that it could include articles that use diverse lexical choices, as well as allow the emergence of those that used other acupuncture techniques.

**Table 1:** Search logic and steps.

STEPS	LOGIC
1	1- acup* AND pediatric chronic pain 2- acup* AND paediatric chronic pain
2	1- acup* AND chronic pain AND child* OR teen* OR adolesc*
3	1- moxi* OR auricul* OR cupping AND pediatric chronic pain 2- moxi* OR auricul* OR cupping AND paediatric chronic pain
4	1- moxi* OR auricul* OR cupping AND chronic pain AND child* OR teen* OR adolesc*

The preferred study design for inclusion was randomized controlled trials, but prospective studies such as single-arm and case series were also accepted. Review articles, cross-sectional studies, letters, book chapters, and editorials were excluded. There was no restriction on publication date or language.

After reading the abstracts, those articles that analyzed acupuncture (considered as the insertion of an acupuncture needle in any part of the body) or related techniques (such as moxibustion, acupressure, cupping, and auricular therapy) as an intervention for the management of chronic pain, of any origin, and that included participants under 18 years of age or did not report the age of the sample in their abstracts, were selected for full reading. This selection was performed by three researchers independently and the disagreements were resolved collectively.

Articles that analyzed separately or exclusively the pediatric population, which was considered up to 22 years old, following the 2017 guidelines of the American Academy of Pediatrics,^22^ and that met the criteria described above, were included in the review. The preferred and mandatory outcomes of the search were those of pain intensity and frequency, but others were also analyzed, such as the impact on quality of life.

The included articles were analyzed for quality using the MMAT - Mixed Methods Appraisal Tool,^23^ in view of the variety of proposed designs. The clinical trial that was selected was confronted with STRICTA - Standards for Reporting Interventions in Clinical Trials of Acupuncture,^14^ as a complementary study quality analysis.

## Results

**Figure 1:**
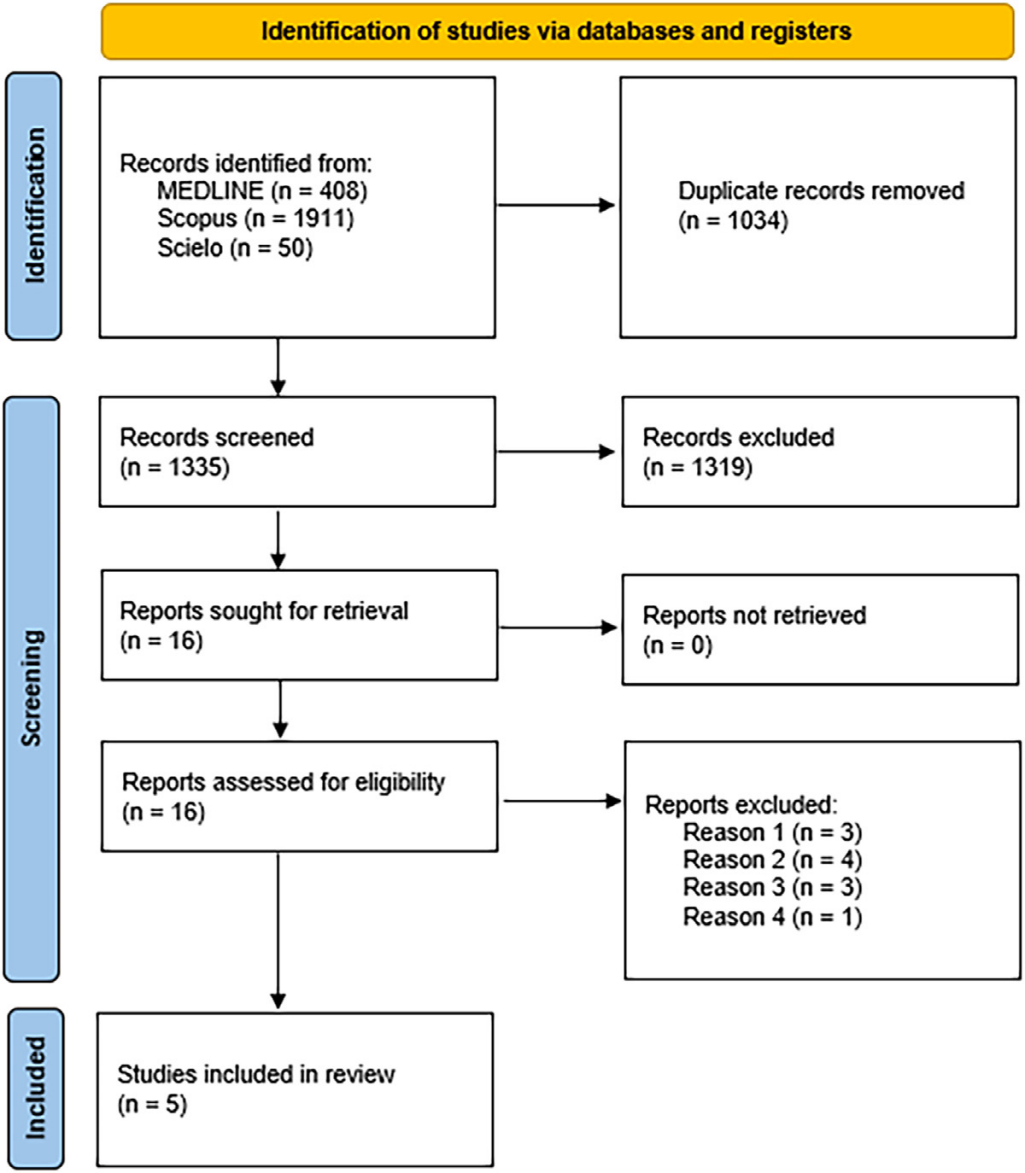
Flowchart (PRISMA).

In total, the searches returned 2369 results (408 in MEDLINE; 1911 in Scopus; 50 in Scielo). After the exclusion of duplicates, 1335 articles remained. These articles were then screened by three researchers (JRB, JURSJ, GPA), who reached a consensus on any disagreements. Applying the selection criteria for study design, target population, and evaluated technique, 16 articles were chosen for full-text reading. Of these, 11 were excluded from the analysis for the reasons explained below, and 5 were included in this review, as shown in the flowchart (Figure 1).

The reasons for the exclusion of the 11 articles that were read in full include:

i. Three articles did not include children under 18 years of age in the study.
ii. Four articles did not provide pain intensity and frequency data separating pediatrics and other age bands in the analysis; authors were contacted to obtain the necessary information, but there was no response.
iii. Three articles did not quantify the improvement, limiting themselves to saying that there was a report of improvement by the patients or family members.
iv. One article did not evaluate the response to pain.

The five articles included in this review have different designs (Table 2), consequently, the quality analysis was performed by MMAT.^23^ The articles were of good quality, except for the lack of description of the randomization procedure in the Randomized Controlled Trial (D).

**Table 2:** Included articles.

	Article	Authors	Country, Year	Study design	Population	Intervention	Comparison	Outcomes
**A**	**A Phase I Study on the Feasibility and Acceptability of an Acupuncture/Hypnosis Intervention for Chronic Pediatric Pain**	Zeltzer LK, Tsao JCI, Stelling C, Powers M, Levy S, and Waterhouse M	USA, 2002	Prospective non-randomized and non-controlled	31 children (19 F, 12 M). Ages between 6 and 18 years (mean 13.0, SD 2.94). All diagnosed with chronic pain	Six 30 min weekly sessions combining acupuncture and hypnosis simultaneously. Acupuncture protocol was individualized following TCM diagnosis criteria, with 20 min of needle retention. Hypnosis conducted a relaxation followed by imagining a safe place and assuming control of the "pain button"	pre to post treatment	Pain intensity measured as "current pain" was reported as reduced by children and parents (*p* < 0,01). 45,2 % of the children reported at least 50 % reduction in "current pain". Pain-related interference also showed a significant reduction (*p* = 0,014) as reported by parents and children.
**B**	**Acupuncture for Pediatric Complex Regional Pain Syndrome**	Lin K and Cynthia Tung C	USA, 2016	Three cases report	Three adolescents (two girls and one boy), ages 11, 12 and 15, with Complex Regional Pain Syndrome	All sessions had a length of 15 min and used manual acupuncture along with electroacupuncture at *ZuSanLi* (ST36). The treatment was completely individualized. C1 underwent 10 sessions in 4 months, C2 6 sessions and C3 5 sessions.	Clinical evolution of the cases	Numerical Rating Scale (NRS) for pain was decreased in all cases (C1 from 6 to 3, C2 from 8 to 6, C3 from 7 to 1). All participants presented improvements in school and sports activities, social life and decreased use of medication.
**C**	**Adolescent Endometriosis-Related Pelvic Pain Treated with Acupuncture: Two Case Reports**	Highfield ES, Laufer MR, Schnyer RN, Kerr CR, Thomas P, and WAYNE PM	USA, 2006	Two cases report	Two adolescent girls with chronic pelvic pain due to endometriosis	C1- 9 sessions over 7 weeks with 20–25 min of needle retention. It was used 10 acupoints per session selected according to the TCM diagnosis. C2 - 15 sessions over 12 weeks with 20–25 min of needle retention. It was used 9 acupoints per session selected according to the TCM diagnosis.	Clinical evolution of the cases	C1 - Pelvic pain reduced from 5 to 8/10 to 3/10. School attendance improved and the patient was able to participate in school groups and field trips, including a 2-month camp. Her analgesic usage also decreased. C2 - Pelvic pain reduced from 8 to 10/10 to a maximum of 4/10. Her mother reported better attention span. The patient was able to participate in a school play and joined the hockey team, indicating less pain interference.
**D**	**Japanese-Style Acupuncture for Endometriosis-Related Pelvic Pain in Adolescents and Young Women: Results of a Randomized Sham-Controlled Trial**	Wayne PM, Kerr CE, Schnyer RN, Legedza ATR, Savetsky-German J, Shields MH, et al	USA, 2008	Randomized controlled trial	18 Females aged 13–22 years old with chronic pelvic pain due to endometriosis. Intervention group: 10 participants Control group: 8 participants	Intervention: 16 sessions of Japanese style acupuncture over 8 weeks. Treatment was individually tailored based on a priori-determined decision algorithm guideline. It included needling 8–12 acupoints, moxibustion on back *shu* points and electro-stimulation on auricular acupuncture points. Control: 16 sessions of non-perforating acupuncture over 8 weeks. It was performed to mimic the active therapy, including the moxibustion and auricular stimulation, but without using acupuncture points.	Compares outcomes inter and intra intervention groups and pre-post treatment. Assessments at baseline, 4weeks, 8 weeks and 6 months following the treatment start.	Pain intensity was better reduced in active acupuncture at 4 weeks assessment (*p* = 0,004). At other assessments there was a tendency, but no statistical significance was achieved. Quality of Life as assessed by the questionaries (EHP, PQoLI) show a trend of improvement in active acupuncture group, but differences between groups were not statistically significant. Pelvic pain-related limitations showed greater improvement in the active treatment group (at 4 weeks the reduction from baseline was 3.4 point against only 0.5 in the placebo [*p* = 0,02]). At 8 weeks and 6 months the difference remained, but between groups comparison showed no statistical significance.
	**The Use of Acupuncture for Pain Management in Pediatric Patients**	Johnson A, Kent P, Swanson B, Rosdil A, Owen E, Fogg L, and Keithley J	USA, 2015	Prospective non-randomized and non-controlled	55 participants (69 % F, 31% M). Ages ranged 7–20 years old. All diagnosed with chronic pain.	8 weekly sessions of manual acupuncture with length of 30 min each. Treatment was completely individualized	pre to post treatment	There was a significant decrease in pain intensity and in Pain Dimensions (all *p* < 0,001). Only 56,4 % completed all eight sessions.

Under the prism of STRICTA,^14^ article (D) fully met the criteria for the preparation and description of a clinical trial of acupuncture, which demonstrates the care in the development of the protocol and its execution. The points that were not reported in the article in question were presented in a separate article, cited in the text, which discusses in more detail the protocol executed.

Due to the methodological differences and design of the included studies, it was not possible to perform a synthetic analysis of the data or to produce a meta-analysis. Thus, this review is based on an individual and descriptive analysis of the studies in conjunction with a comparative discussion between them, as well as the base literature of the proposed theme.

### Study (A)^24^ – Zeltzer et al. (2002), United States of America

Article (A) performs a non-controlled prospective feasibility study of treatment with concomitant Acupuncture and Hypnosis, in a population of 31 children (6–18 years old), with various diagnoses of chronic pain (46 % headache, 21 % abdominal pain, 11 % fibromyalgia, 11 % complex regional pain, 4 % juvenile rheumatoid arthritis, 4 % back pain and 4 % chest pain).

The protocol consisted of six weekly acupuncture sessions with hypnosis during the needle retention time. The choice of points used followed the syndromic diagnosis of Chinese Medicine for each participant and, therefore, was not standardized. The needles were retained for 20 min in each session, and, during this period, a standardized hypnotic imagination process was conducted. Participants were asked to imagine their brain as a control center similar to an airplane, where there would be various buttons and levers. Then, they were guided to find the control of the region of the body that bothered them and adjust the intensity to the correct value.

Pain was assessed by The Varni-Thompson Pediatric Pain Questionnaire (V-T PPQ),^25^ which was completed pre and post-treatment. This questionnaire uses a 0–6 scale in conditions that analyze the intensity of pain and its impact on quality of life. Using the paired *t*-test to assess the responses before and after the intervention, the study showed that both patients and parents reported improvement in pain intensity (*p* < 0.001 for patients and *p* < 0.01 for parents) and in the interference of pain in quality of life (*p* = 0.014 for patients and *p* < 0.01 for parents). The authors also calculated the percentage of patients who reported an improvement in pain intensity greater than or equal to 50 % comparing pre- and post-treatment, reaching the number of 45.2 % of participants.

There was no calculation of effect magnitude or follow-up.

No adverse event occurred to the treatment.

### Study (B)^26^ – Lin and Tung (2016), United States of America

The article (B) presents a retrospective discussion of three cases treated at a tertiary pediatric pain service for complex regional pain syndrome. Patients received six weekly acupuncture sessions (which could be continued if deemed necessary), using Traditional Chinese Medicine as the theoretical foundation for the technique and the choice of points used. In addition to the placement of needles, continuous low-frequency electric stimulation (2 Hz) was used at the ZuSanLi (ST36) point bilaterally. Cupping and GuaSha (complementary TCM techniques) were also used on the backs of the patients. The usual treatments of the service were maintained so that traditional Chinese Medicine was a complementary treatment.

Case 1 is a 15-year-old girl with persistent pain in the left foot for nine months after twisting it. The average pain score (0–10) was 6, with 8 in more intense crises. After six weekly sessions, the patient no longer missed classes due to pain, was participating normally in her activities and the intensity had reduced to an average of 3/10. The sessions were spaced out and in her last appointment, the 10th session four months after the start, she reported an increase in symptoms with the pain returning to 5/10.

Case 2 is an 11-year-old girl also with pain in the left foot due to the presence of an accessory navicular bone. She reported discomfort when practicing ballet and the impossibility of standing on her toes. At the first appointment, she also complained of allodynia in the medial region of the left foot. She assessed her pain as 8 out of 10 points. After the 6th session, the patient reported significant improvement in her daily activities, stopped using topical medication and assessed her pain at an intensity of 6/10.

Case 3 is a 12-year-old boy with persistent pain in his left ankle and foot after a tibial plateau fracture in an accident five months ago. He reported significant limitations in his activities, being unable to play sports and run. The reported pain intensity was 8 out of 10 points. After the third session of acupuncture, the patient already reported that the pain no longer interfered with his daily activities and quantified it at 2/10. In his fifth session, he reported that he had resumed sports, including participating in competitions.

There was no calculation of effect magnitude or follow-up.

There were no adverse effects to the treatment.

### Study (C)^27^ – Highfield et al. (2006), United States of America

The article (C) presents the treatment with acupuncture and moxibustion of two cases of chronic pelvic pain related to endometriosis in adolescents. The frequency and number of sessions varied. The rationality of TCM was used for the choice of points and stimulation technique. Pain was assessed by a Numerical Rating Scale ranging from 0 to 10.

Case 1 underwent a course of nine sessions in seven weeks and showed important clinical improvement (initial pains were assessed from 5 to 8 and, after treatment, they were assessed as 3), being able to take a two-month trip to a camp, due to less frequent and intense pain, reporting a decrease in the use of analgesic medication. Upon returning, she resumed sessions with a fortnightly frequency. After one year of treatment, the patient improved her school attendance, being able to participate in extracurricular groups as well.

Case 2 underwent a course of 15 sessions in 12 weeks. At the beginning of the treatment, she reported pains of 7 to 8, with daily frequency, and 10 during the menstrual period. Throughout the treatment her pain improved, her school attendance increased, and she was able to participate in a school play. At the end of the treatment, she had only occasional pains, with a maximum intensity of 4 out of 10 points.

There was no calculation of effect magnitude or follow-up.

There were no adverse effects to the treatment.

### Study (D)^28^ – Wayne et al. (2008), United States of America

The article (D) is a randomized, double-blind, placebo-controlled clinical trial that evaluates an acupuncture intervention in the management of chronic pelvic pain related to endometriosis in adolescents. The primary outcome was changes in pelvic pain as measured by the Endometriosis Symptoms Severity Scale ^29^ but were also analyzed Quality of Life outcomes and measurement of the serum concentrations of IL-6 and TNF.

18 adolescents, aged 13–22 years, were randomized (10 to intervention and 8 to control). Both groups received 16 sessions in 8 weeks, with the intervention group receiving Japanese acupuncture the control group receiving non-perforating sham acupuncture, and a follow-up 8 weeks and 6 months after treatment.

Pain intensity was reduced in the intervention group compared to the control group at 4 weeks (*p* = 0.004), but after 8 weeks and six months, the difference between the groups was not statistically significant. There was no difference between the groups in the concentration of inflammatory markers in the blood. The calculated magnitude effects were considered very significant on the impact of pain and significant on quality of life.

The reported adverse effects were mild, such as local pain and a feeling of lightheadedness immediately after the session.

### Study (E)^30^ – Johnson et al. (2015), United States of America

The study (E) is a single-arm trial that uses the Adolescent Pediatric Pain Tool (APPT) ^31^ to evaluate the pain symptoms and other Quality of Life instruments to assess the effects of an 8-session acupuncture intervention on chronic pain and quality of life of patients. The study followed the logic of Traditional Chinese Medicine to perform treatments that were completely individualized according to the syndromic diagnoses performed.

55 participants, aged 7–20 years, entered the study. The chronic pain complaints varied as follows: headache (18 %), musculoskeletal (75 %) and abdominal/pelvic (7 %). Only 56.4 % of the patients completed the eight sessions, with the reasons for not completing interference with work/school hours, and difficulty with distance and four patients died in the middle of the treatment due to their underlying diseases. No adverse effects were reported to the treatments.

The article shows a progressive reduction in pain throughout the sessions and a significant reduction in the pain intensities reported. However, there is no evaluation of the clinical impact of this numerical effect beyond its statistical relevance (*p* < 0.001).

There was no follow-up.

### Comparative description

Altogether, these articles (two case series, one of three and one of two cases, two single-arm studies) comprise 86 participants and only one randomized clinical trial with 18 patients (all female teenagers). In addition, only one study performed follow-up and quantified the magnitude of the clinical effect. A variety of acupuncture interventions are noted, from Chinese and Japanese techniques to the application of other TCM therapies such as cupping and moxibustion.

Beyond that one study performed acupuncture with hypnosis simultaneously. No significant adverse effects were reported by the articles.

## Discussion

The present findings reinforce the perception of a small number of clinical studies that evaluate the effect of acupuncture in the management of chronic pain in the pediatric population.

The articles analyzed showed positive results in terms of immediate clinical response, including improvements in quality of life, school participation and social interaction. These are significant impacts for patients who suffer from chronic pain. However, the maintenance of these effects over the long term was not observed in the follow-up of the clinical trial.

The two case series articles (B and C) bring an interesting perspective from a qualitative standpoint. Both describe improvement in school attendance, participation in social and sporting events, and giving patients back the characteristics of childhood that they were losing. They thus demonstrate the individual clinical relevance of acupuncture and the impact that this treatment has on the patients. Further studies should focus on the use of social-educational outcome measures.

The single-arm articles (A and E) showed a larger number of participants and achieved statistical significance of the differences pre- and post-treatment, providing evidence for the clinical use of this technique in cases of pediatric chronic pain. As a strength, these studies included various diagnoses, which are more in line with the IASP recommendations and suggest a broad action of acupuncture on pain mechanisms. However, a major bias present in study A is the concomitant use of hypnosis during acupuncture sessions, as it prevents the separation of the effects of each of the techniques.

The only randomized controlled trial included (D) was placebo-controlled. Surprisingly, effect size indicated a “very significant” effect in pain reduction, but it disappeared at the eight-week follow-up, suggesting that treatment benefit was not persistent. A limitation of this study is its small sample, with the authors stating that the initial calculation was 42 participants, not being able to reach half that value. In addition, the study assesses only chronic pelvic pain due to endometriosis, which cannot be generalized to other forms of chronic pain.

How to conduct a control intervention is an important discussion in current acupuncture research. There are many ways to produce sham-acupuncture, such as needle outside acupoints, minimal insertion of the needle, and non-insertion of needle.^32^ But, as physiological studies advance, invasive sham-acupuncture show some clinical effects. Therefore, the current evidence suggests that non-invasive needling is the best control for acupuncture but is not well established that is in fact a true placebo.^32^

This study (D) also performs the dosage of inflammatory mediators in the blood of the participants, finding no statistical difference in the samples. Other studies reached similar conclusions evaluating dysmenorrhea,^33^ which suggests that the analgesic effects of acupuncture in these cases do not occur through anti-inflammatory pathways.

Despite integrative health practices being widely used in children and teenagers,^9^^,^^34^ clinical studies investigating their efficacy in the management of chronic pain in this population are still preliminary.

Given the importance of chronic pain in pediatrics, both for its prevalence and for the impact it has on patients and families,^2^^,^^3^ this systematic review clearly points to the scarce number of scientific intervention studies, in addition to the variety of methodologies and techniques used. It supports the need for further investigation of the topic.^35^^,^^36^

For example, in study (A), 94 % of the invited patients agreed to participate in the study and 90 % of the participants completed the sessions. In study (D), 77.8 % of the participants completed the 16 sessions. In article (E), only 56.4 % completed the proposed treatment, but the reasons for abandonment did not include non-acceptance or adverse effects, only the difficulty of locomotion and conflict with school and work. This suggests that acupuncture is well tolerated by children and adolescents, which is in line with other articles found in the literature^36^, ^37^, ^38^ and can be part of multi-professional protocols for the management of chronic pain in the pediatric population.

In addition, there were no serious adverse events reported in the studies included. Only article (D) reports cases of mild events such as pain at the puncture site, bruises, lightheadedness, and dizziness after the sessions, all of which were transient and without major complications. Knowing that these studies demonstrated care in their execution, with trained and experienced professionals, the practice of acupuncture seems to be safe, strengthening what has already been pointed out in the literature.^39^^,^^40^

In general, the studies included in this systematic review attest to the potential of acupuncture in the symptomatic management of patients with chronic pain, whose effects were not restricted to reducing pain intensity and frequency, but also in the impact of pain on daily activities, such as attending school, and on quality of life. This finding adds evidence that acupuncture seems to be a promising and safe method and the possibility of its inclusion in pediatric pain teams deserves more attention.

However, the scientific strength of this data is still incipient. Given the biases and limitations of these articles, such as the small sample size, the lack of controls, the variety of techniques used, and the lack of follow-up. Even so, it presents a therapeutic possibility, since article (A) showed that 45 % of the participants reported at least 50 % improvement in pain and study (D), which has the best design and adequate methodology as assessed by the tools used, showed a "very significant" magnitude of effect in pain management in a 4-week analysis.

Despite the positive response observed in the studies, the only study with follow-up did not find statistical significance in the follow-up from 8 weeks, which suggests that acupuncture may have a short-lasting effect on chronic pain in pediatrics. Article (C), in its two cases, resulted in cumulative and long-term improvement, as its follow-up lasted for almost a year. This divergence may be due to the methodological differences between the studies, including the proposed treatment, since (D) uses Japanese acupuncture and (C) Chinese acupuncture. In any case, this study aims to investigate the effect of acupuncture methods on pediatrics pain management rather than to directly compare types of acupuncture methods.

This lack of standard in studies involving acupuncture is a present difficulty that has already been discussed in previous articles,^36^ which originates in its long history and practice by various peoples. So, it is necessary to carry out better studies, with larger samples and control groups, to determine the indications of each technique, and its limitations and increase the certainty of the positive clinical findings.

A limitation of research, including reviews, carried out in the West is the difficulty of access and use of Chinese biomedical databases. This has been discussed in more recent articles, including topics that go beyond Traditional Chinese Medicine, but the authors observed a limitation in access and low frequency of use of these databases.^41^ Possibly the inclusion of these databases would bring different results in this research, such as more clinical trials and bigger samples, knowing the size of the Chinese population and the prevalence of acupuncture usage in this country. But its execution was not possible.

Another issue that needs to be pointed out is the choice of the term chronic pain. A general term (chronic pain) was adopted to search broadly for various diagnoses, following the current recommendations. However, this choice may have impacted the search, as some articles may use specific terms in their descriptions such as dysmenorrhea, headache and fibromyalgia and may not have appeared in the surveys. At the same time, it would not be feasible to search for all types of chronic pain individually and, given the lack of studies, this broader strategy was chosen.

The studies provided weak scientific evidence to justify the use of acupuncture in children and adolescents with chronic pain, from an interdisciplinary perspective. Although the included studies are favorable to the usage of acupuncture, there are many limitations in their execution.

For example, the main one being the variability of techniques involved in the treatments, such as the concomitant use of hypnosis in acupuncture sessions and other Chinese Medicine techniques, without standardization of therapeutic conduct. Another major limitation is the lack of control groups, which considerably decreases the strength of the results obtained. Only one of the studies performed a group comparison.

In general, there is scant and weak scientific evidence to support the systematic use of acupuncture in the management of chronic pain in pediatrics. However, it seems to be a promising adjuvant therapy, with good clinical impact and no significant adverse effects. However, due to the limited number of articles available and the heterogeneity in their designs and therapeutic proposals, it is not possible to conclude definitively about the clinical relevance of these findings. More studies, especially controlled clinical trials, are needed to validate this hypothesis.

## Conflicts of interest

The authors declare no conflicts of interest.
